# Performance of RAFT-based and conventional bulk-fill composites cured with conventional and high irradiance photocuring: a comparative study

**DOI:** 10.1186/s12903-025-06046-7

**Published:** 2025-05-13

**Authors:** Noura Arafa, Dalia I. Sherief, Lamia M. Elmalawanya, Mohamed S. Nassif

**Affiliations:** 1 Biomaterials Department, Faculty of Dentistry, Ain-Shams University, African Union Organization Street, Cairo, Abbasia 11566 Egypt; 2 Badya University, Badya City, Giza, Egypt

**Keywords:** RAFT polymerization, Ultra-fast curing, Bulk-fill composites, Mechanical properties, Polymerization shrinkage, Degree of conversion

## Abstract

**Background:**

Understanding the impact of advanced photocuring and composite formulations for clinical outcomes and restoration durability. This study evaluated the degree of conversion (DC), polymerization shrinkage strain, and flexural properties (Flexural strength and modulus) of conventional and RAFT-based bulk-fill resin composites cured with conventional and high-irradiance ultra-fast photocuring.

**Methods:**

A total of 80 specimens of a RAFT-based bulk-fill resin composite (Tetric PowerFill, TP, Ivoclar Vivadent AG Bendererstrasse 2 9494 Schann/Liechtenstein) and a conventional bulk-fill composite (Tetric N-Ceram, TN, Ivoclar Vivadent AG 9494 Schaan/Liechtenstein) were cured using two protocols: high irradiance ultra-fast mode (2700 mW/cm^2^ for 3 s) and conventional mode (900 mW/cm^2^ for 20 s). The DC was measured using FTIR Spectroscopy(Thermo-Nicolet 67,000, USA), and the polymerization shrinkage strain was quantified with a polyimide-backed electrical resistance strain gauge using a strain meter (PCD-300A Kyowa-Electronic Instruments Co, LTD, Tokyo, Japan). Flexural strength σ_f_ (MPa) and modulus E_f_ (MPa) were assessed using 3-point loading in a universal testing machine (Instron 3365, Norwood, MA, USA, with a maximum load capacity of 5 kN) immediately after curing and after thermal aging (10,000 cycles). Results were analyzed using multi-factorial ANOVA with a significance level set at (*p* ≤ 0.05).

**Results:**

The DC for TP showed no significant differences between curing modes, with values of 57.82% in fast mode and 55.3% in conventional mode. Similarly, its mechanical properties remained relatively consistent, with σf measuring 121.66 MPa in fast mode and 137.5 MPa in conventional mode, while the Ef was 6078.50 MPa and 6167.26 MPa, respectively. In contrast, TN exhibited a lower DC in fast curing (50.27%) compared to conventional curing (61.5%). However, its mechanical properties remained nearly unchanged, with σf recorded at 135.34 MPa in fast mode and 137.26 MPa in conventional mode, and Ef at 6356.54 MPa and 6857.2 MPa, respectively. Moreover, TP showed greater resistance to mechanical property degradation after thermal aging compared to TN.

**Conclusions:**

The RAFT-based bulk-fill composite performed comparably to the conventional composite in both curing modes while demonstrating greater durability. However, fast curing of the conventional bulk-fill composite resulted in unacceptable properties, underscoring the importance of selecting appropriate materials and curing protocols to ensure long-lasting restorations.

## Background

Dental resin composites are widely used in restorative dentistry due to their aesthetic appeal, ease of application, and ability to bond to tooth structure. These materials offer several advantages, such as excellent translucency, wear resistance, and the ability to be sculpted directly in the cavity [1, 2]. Although resin composites are widely used as aesthetic restorative materials in modern dentistry, they have several limitations that can compromise the longevity and success of the restoration if not managed appropriately. Common issues include water sorption, solubility, secondary caries, wear, color instability, postoperative hypersensitivity, marginal or bulk fractures, polymerization shrinkage, and the associated stresses [3].

One of the main drawbacks of dental resin composite is the polymerization shrinkage (PS) resulting from the composite shrinkage after polymerization. The PS can compromise the adhesive-composite interface, potentially leading to marginal gaps, which may contribute to marginal staining and, over time, secondary caries. Also, polymerization shrinkage stress(PSS) resulting from PS might cause cracks or fractures in the adjacent dental structures and cuspal deflection [4, 5].

Beyond polymerization shrinkage, other critical properties, such as flexural strength, modulus of elasticity, and the degree of conversion, play a significant role in the durability and mechanical performance of these materials [6].

Modifications in the photocuring protocols were developed to allow for a more viscous flow of the resin composite before the majority of PSS started to build up [7–9] Moreover, conventional resin composites are placed in small increments to decrease the C factor which subsequently decreases the PSS [10]. However, this application protocol made composite restorations more technique-sensitive and time-consuming, which is uncomfortable for both patients and clinicians.

This leads to the development of bulk-fill composites with high translucency thus allowing the transmission of light so that larger increments (4 mm) of composites can be placed and, composed of flexible monomers with a high molecular weight to decrease the PSS and ensure a proper degree of conversion (DC). In addition to minimizing shrinkage, bulk-fill composites were designed to improve clinical efficiency, enhance ease of application, and achieve a higher degree of conversion, making them a versatile and practical option for dental restorations" [11].

Fast high irradiance light curing (3 s)(fast curing) of bulk-fill resin composites was introduced lately to further save the patient's chair time, also, shorter curing times reduce non-material factors like contamination and inattention, highlighting the significance of light curing in dental practice [12]. Such a curing technique relies on the concept of exposure reciprocity which one could deliver the required photon dose over a shorter irradiation period by significantly increasing the light irradiance (photons/second) [13]. This was achieved first by adding Norrish type I monoacylphosphine oxide photoinitiator, namely Lucirin-TPO to the common ketone camphorquinone (CQ)/amine photoinitiation system, and also by choosing appropriate irradiation parameters (wavelength range, irradiance and irradiation time) [14–16]. The type I monoacylphosphine oxide photoinitiator has higher photon absorption and higher cleavage ability compared to CQ so it gives a higher concentration of free radicals resulting in a higher DC with less curing time [16]. The fast-curing concept (3 s) has been questionable regarding its effect on PSS which might negatively affect the mechanical performance of the resin composite restorations and the adhesive layer [16].

A new modification in the chemistry of resin composites by which the free radical polymerization mechanism was shifted into Reversible Addition Fragmentation chain Transfer (RAFT) polymerization was achieved by the addition of thiocarbonyl thio chain transfer agents [13, 16, 17]. RAFT polymerization is a type of dynamic covalent chemistry [18] that gives a new pattern of crosslinked polymers called covalent adaptable networks which can adjust their internal structure through two sets of reactions; pre-equilibrium and the main equilibrium in addition to the conventional radical polymerization steps. This results in a polymer with a more homogenous polymer network. First, the radical is transferred from the initiator molecules to the monomer units, followed by the propagation step to increase the chain length. The RAFT pre-equilibrium step follows, in which the propagating radical reacts with the RAFT agent to form an intermediate radical (RAFT-adduct radical). This RAFT-adduct radical can then undergo a fragmentation reaction in the pre-equilibrium and main equilibrium steps [2, 16, 19] which is called rate retardation. This results in a delayed increase in the molecular weight (MW) and cross-linking compared to the free radical polymerization, and ultimately, the PSS can also be decreased. So RAFT polymerization is particularly relevant in high-irradiance curing due to its ability to regulate polymerization kinetics, reducing shrinkage stress and enhancing mechanical properties.

However, the potentially decreased overall crosslinking density may impart poorer mechanical properties due to the decrease in the MW which is associated with the high fragmentation rate. But, this is less likely to occur when low thiol concentrations (up to 10 wt%) [2] are added to delay the gelation thus reducing the stress without prejudice to final mechanical properties. The fragmentation rate constant is controlled by the concentration of the RAFT agent and the formed radicals during the initiation step [2] which depends on the irradiance of the light cure unit [19].

A study by Garoushi et al. [1] investigated the effect of ultra-fast curing on a conventional resin composite (Essentia U) and found that it yielded inferior results. This dictates the need to study the effect of ultra-fast curing on other types of conventional composites with different chemical compositions including the types of the used initiators and the inorganic fillers content.

Additionally, a study conducted by Ilie et al. [13] investigated different light curing protocols (3 s and 10 s) on a RAFT-based bulk-fill composite (Tetric PowerFill) regarding its DC, flexural strength, and modulus of elasticity compared to a conventional bulk-fill composite (Tetric Evo-Ceram) cured for 10 s which used as a control. Tetric PowerFill exhibited comparable properties under both curing protocols. However, there is no evidence in the literature regarding the effect of aging on the degradation of the properties of RAFT-based composites.

Thus, this study was conducted to investigate the effect of conventional photocuring (20 s) and high irradiance ultra-fast photocuring (3 s) on a fast-curing bulk-fill composite (modified with RAFT agent) and a conventional bulk-fill composite regarding the degree of conversion, polymerization shrinkage, flexural strength, and modulus of elasticity. The effect of aging on flexural properties was also tested. While rapid curing has been shown to compromise conventional composite [20, 21], we included a conventional bulk-fill composite to compare its performance under identical conditions. This allows for a direct assessment of whether RAFT technology can overcome the known limitations of conventional formulations under high-irradiance curing.

The null hypotheses tested were that 1- the change in the curing protocol (high irradiance 3 s/conventional 20 s) of a fast curing bulk-fill (RAFT-based) would not affect its degree of conversion, polymerization shrinkage, and flexural properties before and after aging. 2- the change in the curing protocol (high irradiance 3 s/conventional 20 s) of a conventional curing bulk-fill would not affect its degree of conversion, polymerization shrinkage, and flexural properties before and after aging.

## Materials and methods

A fast-curing bulk-fill resin composite modified with RAFT polymerization (Tetric PowerFill, Ivoclar Vivadent AG Bendererstrasse 2 9494 Schann/Liechtenstein) and a conventional bulk-fill composite (Tetric N-Ceram, Ivoclar Vivadent AG 9494 Schaan/Liechtenstein) were used (Table 1).

**Table 1:** Materials investigated

Trade name	Resin Matrix*	Filler load % (vol)*	Photo-initiating system
**Tetric PowerFill**	Bis-GMA Bis-EMA UDMA Bis-PMA DCP the organic matrix modified with a chain fragmentation agent (β-allyl sulfone)	53–54 vol% Barium glass, copolymer, Ytterbium trifluoride, and Si-Zr mixed oxide particle size (0.11µm and 15.46 µm)	(CQ)/amine, and Ivocerin (bis-(4-methoxy benzoyl)diethyl germane)
**Tetric N-Ceram Bulk Fill**	Bis-GMA Bis-EMA UDMA	53-55vol % Barium glass, prepolymer, Ytterbium trifluoride, and mixed oxide particle size (0.04µm and 3µm)	(CQ)/amine, an acyl phosphine oxide (TPO), and Ivocerin (bis-(4-methoxy benzoyl)diethyl germane)

*Matrix Monomers

*Bis-GMA: bisphenol-A-diglycidyl dimethacrylate*

*Bis-EMA: bisphenol-A-polyethylene-glycol-diether dimethacrylate*

*UDMA: urethane dimethacrylate*

*Bis-PMA: Propoxylated Bisphenol A dimethacrylate*

*DCP: Tricyclodecane-dimethanol dimethacrylate*

^*^Filler percentages and type as reported by the manufacturer

CQ camphorquinone

A light emitting diode (LED) curing unit (X-cure, Guilin Woodpecker Medical Instrument Co., Ltd.L2140088X) was used for photocuring in two different curing modes (high irradiance ultra-fast curing mode with 2700 mW/cm^2^ for 3 s and conventional mode with 900 mW/cm^2^ for 20 s)both were continuous curing mode. The intensity of the light-curing source was checked using an LED radiometer (Model 100 curing radiometer, Kerr, USA).

**Fig. 1:**
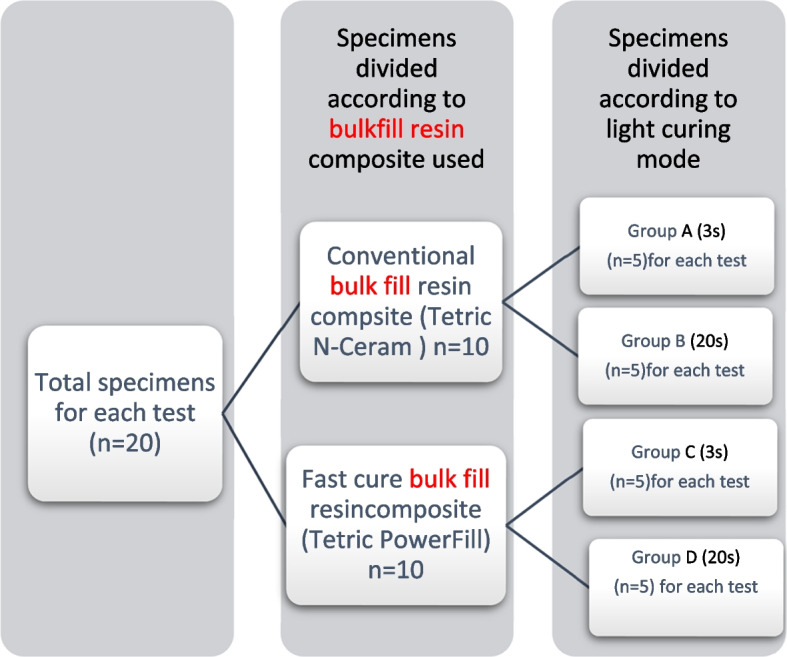
Specimens grouping

A total of 80 specimens of the above-described resin composites were measured for the two different curing modes for all tests, 20 specimens for each degree of conversion (DC%) and polymerization shrinkage strain (Fig. 1) and 40 specimens for Flexure strength was measured for both materials as a function of curing mode and aging time.

The specimens were prepared in controlled laboratory conditions (temperature typically between 20–25 °C and a consistent relative humidity) to ensure standardization Specimens were stored in sealed containers to avoid changes in moisture content that could affect material properties.

### Degree of conversion

A total sample size of 20 specimens (*n* = 20) was divided into 4 groups (*n* = 5 for each group) (Fig. 1). Specimens were prepared in a split stainless steel mold of 3 mm diameter and 1 mm thickness. The resin composites were packed into the mold, covered with celluloid strips, and then lightly pressed with a glass slide to remove the excess material. Ten specimens (5 specimens of each composite resin) were cured by the fast mode with 2700 mW/cm^2^ for 3 s, and 10 specimens (5 specimens of each composite resin) were cured by the conventional mode with 900 mW/cm^2^ for 20 s. Specimens were removed from the mold and excess material was removed using a #600 grit silicon carbide (SiC) paper. The specimens were stored dry in an incubator at 37℃ ± 1 within a light-proof container filled with silica gel to be tested after 24 h. The degree of conversion (DC%) was measured using Fourier Transform Infrared (FTIR) spectroscopy (Thermo-Nicolet 67,000, USA) [13, 16, 22, 23]. To ensure accurate and reproducible FTIR spectroscopy results, it was calibrated by performing a background scan of a blank reference (potassium bromide for KBr pellet method). Each cured specimen was ground from the surface into powder. A small amount of the powdered specimens was then mixed with Potassium Bromide (KBr) powder salt. The amount of the powdered specimen was about 2% of the KBr amount. The mixture was ground for 3 to 5 min and then placed into a pelleting device followed by pressing in a hydraulic press with a load of 8 tons to obtain a pellet. This pellet was then placed in a holder attachment within the spectrometer for testing. Each specimen was scanned three times and the average of the three readings was calculated. Uncured specimens for each composite type were smeared onto a thin KBr disc and then placed in the holder attachment of the spectrometer for testing.

**Fig. 2:**
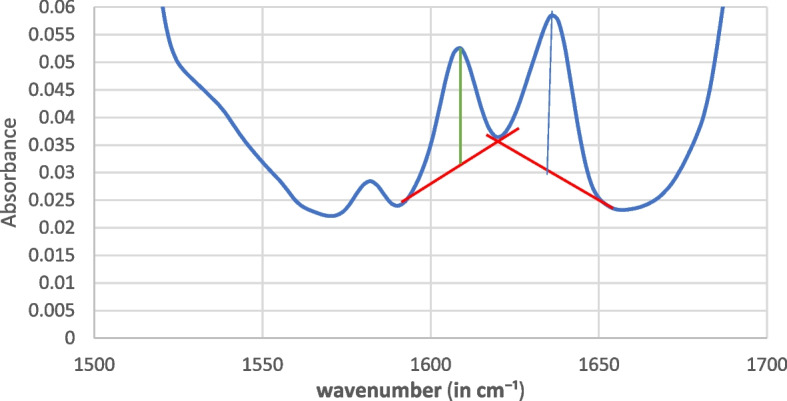
Schematic representation of the baseline method used to determine the ratios of the absorbance peaks corresponding to the aliphatic (1638 cm-1) and aromatic (1608 cm-1) C = C bonds for a composite material. The peak heights were measured in relation to the baseline (red line)

The DC% was measured by computing the variation in the ratio of the absorbance intensities of the aliphatic carbon to carbon (C = C) double bond peak at 1634 cm^−1^ by employing the aromatic C = C double bond peak at 1608 cm^−1^ as a wavenumber of the reference spectral band polymerization of the uncured material (using the baseline method) as presented in Fig. 2 [24, 25].

Two baseline points were chosen on either side of the peaks which represent regions where there is minimal or no absorption from the sample. The intensity of the peaks was recalculated relative to this new baseline.

The DC% was calculated for each sample using the following equation [23, 26]:

$$\text{DC}\%= [1-(\text{a}/\text{ b}) ]\text{ x }100$$

where .0

a = (aliphatic absorption peak/aromatic absorption peak) polymer.

b = (aliphatic absorption peak/aromatic absorption peak) monomer.

### Polymerization shrinkage strain

**Fig. 3:**
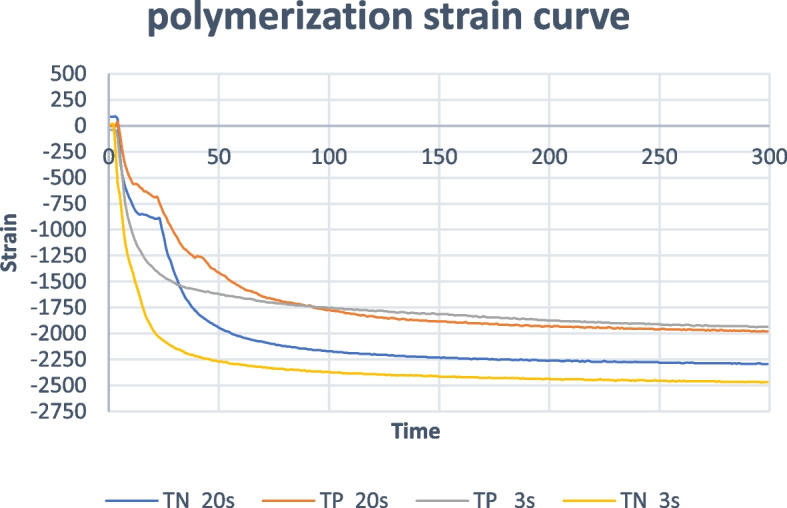
Strain versus time curves for the two composites cured with different curing modes

Polymerization shrinkage (PS) strain was measured for each group (*n* = 5) using a polyimide-backed electrical resistance strain gauge (Kyowa Electronic Instruments Co, LTD, Tokyo, Japan). The strain gauge was placed on the bottom at the center of a Teflon white split mold of 6 mm in diameter and 1 mm in thickness, then the resin composite material was packed into the mold and covered by a celluloid matrix. Ten specimens (5 specimens of each composite resin) were cured by the fast mode with 2700 mW/cm^2^ for 3 s, and 10 specimens (5 specimens of each composite resin) were cured by the conventional mode with 900 mW/cm^2^ for 20 s while the gauge wire was connected to the strain meter (PCD-300 A Kyowa-Electronic Instruments Co, LTD, Tokyo, Japan). Before light curing of the specimen, the strain gauge system was balanced to zero to eliminate any offset and ensure that the initial readings start from a baseline with no residual strain. PS strain measurement for each specimen was recorded during curing and 5 min following the light irradiation. Strain measurements were conducted using a PCD-300 A data logger (Kyowa Electronic Instruments Co., Ltd., Tokyo, Japan), paired with DAS-100 A data acquisition software for real-time data recording and analysis. Strain values were continuously monitored and recorded in real-time using DAS-100 A’s graphical interface, and then the recorded strain data was performed in DAS-100 A for baseline correction and noise reduction. Further statistical and graphical analysis was conducted using Excel. Strain versus time curves for the different testing conditions were obtained using a strain meter Fig. 3 [27, 28].

### Flexural strength (FS) and flexural modulus (FM)

A total sample size of 40 specimens (*n* = 40) was divided into 4 groups (*n* = 10 for each group). Specimens were prepared using split stainless-steel mold with a central rectangular cavity 12 mm in length, 2 mm in width, and 2 mm in depth. The mold was placed on a glass slide, then after packing the composite material into the mold, was covered with a celluloid strip and pressed lightly with a glass slide. For each group, specimens were divided, one-half of the specimens were tested after 24 h of storage in distilled water in an incubator at 37℃ ± 1 and the second half of the specimens were subjected to 10,000 cycles of thermocycling immersed in distilled water (SD Mechatronic thermocycler THE-1100, Germany) equivalent to 1 year of service in the oral cavity. The thermal cycles were performed between 5 and 55℃ with 15 s dwell time in each bath and a transfer time of 5 s [29, 30]. The flexural strength and flexural modulus were determined in a three-point bending test with a 10 mm distance between the supports. Before testing, specimens were visually inspected for defects such as air bubbles, cracks, or irregularities that could affect the results, and minor surface irregularities were gently polished with #600 grit silicon carbide (SiC) paper to ensure uniform specimen dimensions and prevent stress concentrations during loading. The specimens were loaded until fracture in a universal testing machine (Instron 3365, Norwood, MA, USA, with a load capacity of 5kN) with 0.5 mm/min crosshead speed. The flexure strength and flexural modulus were calculated using Bluehill 3 software using the following equations [31]:

$$\text{F}.\text{S}= 3\text{FL}/{2\text{bd}}^{2}$$

where (F = load at failure in N, L = length between the supports in mm, b = width of the specimen in mm, and d = thickness of the specimen in mm)

$$\text{Ef}={\text{L}}^{3}\text{m}/{4\text{bd}}^{3}$$

where (L = length between the supports in mm,m = Slope of the initial linear portion of the load–deflection curve (N/mm), b = width of the specimen in mm, and d = thickness of the specimen in mm).

### Statistical analysis

Numerical data are presented as mean and standard deviation values. They were checked for normality using Shapiro–Wilk's test and by checking the data distribution. Data were found to be normally distributed and were analyzed using multi-factorial ANOVA. Comparisons of simple effects were done utilizing Bonferroni correction with the pooled error term from the main ANOVA model. The significance level was set at *p* ≤ 0.05 within all tests. Statistical analysis was performed with R statistical analysis software version 4.3.1 for Windows.^1^

## Results

### Degree of conversion results

Mean and standard deviation (SD) values of the degree of conversion (DC%) for different materials are presented in Table 2. There was no statistically significant difference in DC% between the two materials for both fast and conventional modes.

**Table 2:** Mean and standard deviation (SD) values of degree of conversion (%) of different materials for different curing modes

Material	Degree of conversion (DC%) (mean ± SD)		*p*-value
Curing mode	**Tetric PowerFill**	**Tetric N-Ceram**	
**Fast mode**	57.9 ± 8.12	50.3 ± 2.67	**0.127**
**Conventional mode**	55.3 ± 5.47	61.6 ± 6.60	**0.193**
***p*-value**	**0.624**	0.019*	

^*^significant (*p* < 0.05) ns, non-significant (*p* > 0.05)

The effect of curing modes on the degree of conversion is presented in Table 2. Regarding Tetric PowerFill, no statistically significant difference in DC% was found between the conventional and the fast modes while in Tetric N-Ceram, the conventional mode had a significantly higher DC% value than the fast mode.

### Polymerization shrinkage strain

Mean and standard deviation (SD) values of polymerization shrinkage strain for different materials are presented in Table 3. Regarding the fast mode, Tetric N ceram had a significantly higher value of polymerization shrinkage strain than Tetric powerfill while for the conventional mode, there was no statistically significant difference between the two materials.

**Table 3:** Mean and standard deviation (SD) values of polymerization shrinkage strain of different materials for different curing modes

Material	Strain (mean ± SD)		*p*-value
Curing mode	**Tetric PowerFill**	**Tetric N-Ceram**	
**Fast mode**	1990.0 ± 163.29	2378.0 ± 124.73	0.003*
**Conventional mode**	2020.0 ± 230.95	2271.0 ± 246.84	**0.135**
***p*-value**	**0.818**	**0.412**	

^*^significant (*p* < 0.05) ns, non-significant (*p* > 0.05)

The effect of curing modes on the polymerization shrinkage strain is presented in Table 3. For both materials, no statistically significant difference in polymerization shrinkage strain values for the different curing modes.

### Flexural strength

For the immediate flexural strength, in both curing modes, no statistically significant difference between the two materials was found. After aging, regarding the fast mode, there was no statistically significant difference in the flexural strength of both materials, while in the conventional mode, Tetric PowerFill had a significantly higher value than Tetric N-Ceram (Table 4). As for the effect of both curing modes on the immediate and after-aging flexural strength, no statistically significant difference between the two materials was found (Table 4).

**Table 4:** Mean and standard deviation (SD) values of flexural strength (MPa)and modulus of elasticity (MPa) for Both bulk fill composite resins using different curing modes and aging conditions

**Aging**	**Material**	**Flexural strength (MPa) (mean ± SD)**		***p***-value	**Modulus of elasticity (MPa) (mean ± SD)**		***p***-value
	Curing mode	**Tetric Powerfill**	**Tetric N-Ceram**		**Tetric Powerfill**	**Tetric N- Ceram**	
**Non-aged**	**Fast mode**	121.7 ± 14.77	135.3 ± 6.68	**0.096**	6078.5 ± 157.96	6356.5 ± 472.12	**0.247**
	**Conventional mode**	137.9 ± 6.66	137.3 ± 10.58	**0.912**	6167.3 ± 362.45	6857.3 ± 358.46	0.016*
***p***-value		**0.055**	**0.740**		**0.629**	**0.096**	
**Aged**	**Fast mode**	105.4 ± 11.93	90.3 ± 15.68	**0.125**	6627.2 ± 370.65	5802.2 ± 252.00	0.003*
	**Conventional mode**	116.9 ± 9.90	92.7 ± 9.27	0.004*	7308.7 ± 655.96	5766.3 ± 800.68	0.010*
***p*-value**		**0.138**	**0.776**		**0.078**	**0.926**	

^*^significant (*p* < 0.05) ns, non-significant (*p* > 0.05)

Regarding the flexure strength of Tetric PowerFill, in the fast mode, no statistically significant difference was found between the immediate and aged samples, while in the conventional mode, the immediate samples had a significantly higher value than the aged samples. For the Tetric N-Ceram, in both curing modes, immediate samples had significantly higher values than aged samples (Table 5).

**Table 5:** Mean and standard deviation (SD) values of flexural strength (MPa) and modulus of elasticity (MPa) for samples with and without aging

Material	Aging	Flexural strength (MPa) (mean ± SD)		*p*-value	Modulus of elasticity (MPa) (mean ± SD)		*p*-value
	Curing mode	Non-aged	Aged		Non-aged	Aged	
**Tetric PowerFill**	**Fast mode**	121.7 ± 14.77	105.4 ± 11.93	**0.092**	6078.5 ± 157.96	6627.2 ± 370.65	0.016*
	**Conventional mode**	137.9 ± 6.66	116.9 ± 9.90	0.004*	6167.3 ± 362.45	7308.7 ± 655.96	0.009*
**Tetric N-Ceram**	**Fast mode**	135.3 ± 6.68	90.3 ± 15.68	< 0.001*	6356.5 ± 472.12	5802.2 ± 252.00	0.049*
	**Conventional mode**	137.3 ± 10.58	92.7 ± 9.27	< 0.001*	6857.3 ± 358.46	5766.3 ± 800.68	0.024*

^*^significant (*p* < 0.05) ns, non-significant (*p* > 0.05)

### Modulus of elasticity

Regarding the immediate values of modulus of elasticity, for the fast mode, there was no statistically significant difference in the modulus of elasticity between both materials, while in the conventional mode, Tetric N-Ceram had a significantly higher value than Tetric powerfill. After aging, Tetric PowerFill had a significantly higher value than Tetric N-Ceram for both curing modes (Table 4).

As for the effect of curing modes on both the immediate and after-aging modulus of elasticity values, no statistically significant difference between the two materials was found (Table 4).

Regarding the effect of aging on the modulus of elasticity of Tetric PowerFill, in both curing modes, aged samples had a significantly higher value than immediate samples. In contrast, for the Tetric N-Ceram, in both curing modes, immediate samples had a significantly higher value than aged samples (Table 5).

## Discussion

The fast high irradiance photocuring of RAFT polymerization-based bulk-fill resin composites is the most recent trend in dental bulk-fill composite manufacturing due to the increased need to simplify the composite application steps.

Accordingly, two types of bulk-fill composites were used in this study, free radical-based composite (Tetric N-ceram (TN)) and RAFT-based material (Tetric PowerFill (TP)). In the TN, the polymerization starts with the initiation step through three types of photoinitiators; (CQ)/amine, an acyl phosphine oxide (TPO), and Ivocerin (bis-(4-methoxy benzoyl)diethyl germane)) [32], while in the TP through two photoinitiators (CQ)/amine, and Ivocerin (bis-(4-methoxy benzoyl)diethyl germane)) [16, 17]. For both resin composites, polymerization is initiated by blue light irradiation within the wavelength range of approximately 400 to 500 nm, which was applied in the present study through a violet-blue LED light curing unit.

In this study, no significant difference in the DC was detected when TP was tested against TN. In the fast-curing mode, the DC of the TP was not significantly different from the TN. In the TP, it was explained by the rate retardation associated with RAFT polymerization which allowed more activation of the monomers giving higher DC [33–35] Although it was expected that the ultra-fast polymerization (3 s) would cause a decrease in the DC of TN due to the absence of the rate retardation which means reaching the gel point faster without more initiation [7, 36], but it has produced a comparable value to RAFT polymerization. This might be explained by the lower size range of the inorganic fillers (0.04–3µm) than in TP (0.11–15.46 µm) which allows more light transmission [37], in addition to including an acyl phosphine oxide (TPO) Type I photoinitiator that gives a higher concentration of free radicals than (CQ). The resulting DC depends not only on the energy dose (irradiance and time) but also on the responsiveness of the photoinitiator [2, 33, 35], so the retardation step in RAFT polymerization was compensated by an additional co-initiator (TPO) in conventional radical polymerization. This was in contradiction with a study by Garoushi et al. [1] in which they compared RAFT-based resin composite TP with a conventional resin composite (Essentia U), the inferior results of Essentia U with fast cure mode were attributed to the difference in chemical composition (type of initiator and content of inorganic fillers) from the TN used in this study.

A study by Edina Lempel et al. [21] demonstrated that the rapid 3-s curing of another RAFT-based resin composite (Filtek One Bulk Fill) resulted in a lower DC, which was attributed to differences in the initiator system, as Filtek One Bulk Fill contains only CQ. Consistent with our findings, this suggests that ultra-fast polymerization is primarily a function of the initiator system in achieving an acceptable DC. Meanwhile, the RAFT agent plays a crucial role in minimizing PSS, contributing to a more durable restoration. However, their study reported that FOB exhibited a more homogeneous internal structure with reduced porosity and lower monomer elution, enhancing polymer network stability and potentially influencing mechanical durability compared to TPF. This contrasts with our findings, which indicate that TPF demonstrates durable mechanical properties.

Also, the increase in temperature with polymerization reactions has a direct relation with the DC, that the fast polymerization rate in both materials is associated with higher heat generation which leads to more monomer conversion [25, 38].

Also in the conventional curing mode, no significant difference was found in the DC between the two materials as the rate retardation in TP is decreased [2, 19] and the two materials undergo the same polymerization kinetics resulting in a similar DC [16]. The decrease in the rate retardation occurred as it depends on the concentration of radicals formed during the initiation step which is lower in the soft cure leading to a higher propagation rate [19].

Also, no significant difference was detected regarding the DC of TP when cured with either fast or conventional curing mode, which is due to the decrease in the rate retardation in the pre-equilibrium step when it is slowly cured but this was compensated by the longer curing time. These results were in line with the results of a study by Ilie et al. [13].

In contrast, the two curing modes had a significant effect on the DC of TN. There was a significant increase in the DC using the conventional mode, which was due to the filler size and the additional photoinitiator, in addition to the delayed gelation compared to the fast mode which allowed more initiation and less propagation [7].

The minimum acceptable DC for clinical success in dentistry as suggested by some authors Ruwaida Z. Alshalia, et al. [22, 39] is 55%. This means that the DC of TP by both curing modes gave an accepted DC for clinical success while the DC of TN with fast curing is considered not acceptable, as an insufficient DC negatively impacts the physical and chemical properties of resin-based composites (RBCs). Unreacted monomers may leach from the polymer network, potentially irritating surrounding tissues due to their toxic nature [40].

Regarding the results of the PS strain, there was a significant decrease in PS strain in TP compared to TN in the fast curing mode. This might be due to the retardation step in the RAFT polymerization which caused a delay in reaching the gel point and was not present in the radical polymerization.

The PS is a function of monomer conversion, while the PSS is a function of monomer conversion and the propagation rate. The lower propagation rate allows chain mobility which decreases the generated PSS, while the higher propagation rate is associated with the increase in the polymer MW reaching the gel point faster and lower chain mobility with more build-up of PSS [27, 40, 41].

Although the fast curing mode is associated with faster gelation compared to the conventional curing mode, there was no significant difference in PS strain between the curing modes in both materials. Regarding the TP in the fast curing, the fragmentation reaction in the pre-equilibrium and main equilibrium steps caused a delay in the polymer crosslinking which might have caused a decrease in the PSS [19, 34]. While the TN in the fast curing has produced significantly lower DC than in conventional curing mode. This might explain that although fast curing should have caused a higher PS strain in TN, but the resulting lower DC caused a decrease in the PS strain.

The DC and the filler content have an impact on the mechanical properties of the material in terms of flexure strength and modulus of elasticity, also the polymer chain architecture and the physical crosslinking are considered important factors [22, 33, 42].

Regarding the results of flexure strength, TP and TN showed no difference in immediate flexure strength for both curing modes because both materials contain comparable filler loading in terms of volume percentage and no significant difference in the DC.

TN showed a significantly higher modulus of elasticity than TP in the conventional mode, which could be due to the RAFT polymerization, which results in a more homogeneous polymer network but with a lower molecular weight, thus affecting the resin's rigidity [2, 22].

Thermal aging causes internal stress due to differences in the coefficient of thermal expansion especially in materials with heterogenous composition, this thermal stress in combination with water sorption results in the hydrolysis of the silane layer and leads to the deterioration of mechanical properties [29, 43, 44]. Also, this effect of thermal aging is higher when combined with the PS strain built in the composite resin That explains why after artificial thermal aging TP showed significantly higher values in mechanical properties in terms of both flexure strength and elastic modulus than TN. Also in TN the lower filler size compared with TP is combined with a higher filler–matrix interface which is considered an important role in the degeneration of the composite [42].

The polymer microstructure has an impact on the material degradation, as the polymer chains might contain some defects including pores and loops which are formed by primary chain rotation [13, 22, 42]. These defects allow more water sorption which accelerates the degradation [13, 22, 42]. This chain rotation is a function of the initiation rate, the higher initiation rate is associated with less chain rotation which results in a polymer network with lower defects [22]. This might explain why fast-cured TP didn't show degradation in the properties after aging.

This may also explain why fast-cured TN did not differ significantly from conventionally-cured TN. It was expected that the conventional curing of TN would result in higher mechanical properties due to its significantly higher DC. However, the fast curing process may have produced a lower network defect, leading to a reduced degradation rate and thus comparable mechanical properties [22]. So the first hypothesis was accepted and the second one was rejected.

However, while rapid curing can increase crosslinking density, it may also reduce microhardness and restrict polymer chain mobility, thereby impacting overall polymerization. This effect leads to a significant reduction in initial microhardness (11–48%) compared to conventional curing, primarily due to increased bimolecular termination, which hinders polymerization [45]. Incorporating RAFT agents, however, provides better control over network formation by maintaining an equilibrium between active and dormant chains. Nevertheless, the micromechanical properties remain highly dependent on the material composition and curing conditions, emphasizing the need for careful optimization.

Also, a study by Matej Par et al. [46] found that rapid high-intensity light-curing negatively affected the marginal integrity of bulk-fill composites, particularly flowable types, due to greater shrinkage and lower strength. Marginal integrity was more influenced by the composite material and thermo-mechanical loading than by curing protocol alone. While conventional curing generally performed better, especially for Tetric PowerFlow in dentin, the effect was material-dependent. Rapid curing should be used cautiously with flowable composites until further clinical validation.

A study by Edina Lempel et al. [21], previously mentioned, aligned with our findings regarding the DC. However, their study reported that FOB exhibited a more homogeneous internal structure with reduced porosity and lower monomer elution [21, 40], enhancing polymer network stability and potentially influencing mechanical durability compared to TPF. This contrasts with our findings, which indicate that TPF demonstrates durable mechanical properties.

The results indicate that RAFT-based composites performed similarly across both curing modes while exhibiting greater durability, making them a viable option for clinicians seeking to enhance restoration longevity. Furthermore, the findings emphasize that fast curing of conventional bulk-fill composites resulted in unacceptable mechanical properties, highlighting the need for careful material selection and optimized curing protocols. This reinforces the clinical importance of aligning composite formulations with appropriate curing strategies to minimize the risk of mechanical failure and excessive shrinkage stress.

The 3 s curing gave an acceptable and durable resin composite restoration but the clinical tolerance for 3 s irradiance should be limited to an exposure distance of 5 mm and angulation of the LCU should be avoided [13, 16], which might not be applicable in the clinical situation. That’s why it is recommended to increase the curing time up to 6 s with high irradiance [16].

The potential impact of heat on the pulp remains a critical concern. Studies have reported that pulpal tissue can tolerate a maximum temperature increase of 5.5 °C, beyond which irreversible pulpitis may occur [47, 48]. The 3-s curing protocol is associated with increased temperature due to the high levels of radiant emittance and the exothermic nature of rapid polymerization.

A study by Samille Biasi Miranda et al. [48] compared the effects of different bulk-fill composite resins and light-curing modes on transdentinal temperature and cell viability. Their findings confirmed that the 3-s high-intensity protocol resulted in a greater temperature increase than the moderate-intensity 10-s standard, demonstrating a direct relationship between irradiance and heat generation. However, despite the temperature rise, both curing protocols showed comparable cell viability.

Given these findings, clinicians should exercise caution when using the 3-s high-intensity curing protocol, particularly in cases where minimal remaining dentin may provide insufficient thermal protection for the pulp [47, 48]. Further research is needed to establish safe and effective curing protocols that balance polymerization efficiency with pulpal health considerations.

Also, studies have claimed that the RAFT polymerization-based bulk-fill resin composites have sufficient opacity with acceptable depth of cure, [16, 42] so comparing the translucency of TP with other bulk-fill composites should be considered.

One limitation of this study is that it was conducted in vitro, which does not fully replicate the complexities of the oral environment, such as variations in temperature, humidity, occlusal forces, angulation of the LCU, and aging effects from factors like salivary enzymes and pH fluctuations. Additionally, while our study assessed mechanical properties immediately after curing and aging, the long-term clinical performance, including wear resistance and marginal integrity, remains uncertain. Future research should focus on long-term clinical trials to validate these findings in real-world conditions.

The use of a hand-held radiometer to monitor the curing unit's light output, rather than a calibrated spectrometer, may limit the accuracy of irradiance measurements. Future studies should employ calibrated devices for more precise control.

## Conclusions

Within the limitations of this in-vitro study, the following could be concluded:

1. Under the conditions of this study, Tetric PowerFill (RAFT-based composite) exhibited acceptable properties and durability following ultra-fast high irradiance photocuring, while the tested conventional composite did not achieve the minimum clinically acceptable degree of conversion
2. The RAFT polymerization-based used in our study resin composite provides comparable mechanical properties to the radical polymerization-based composite resin and is more resistant to degradation.
3. Under the conditions of this in vitro study, Tetric PowerFill (RAFT-based) showed consistent performance across both curing protocols and demonstrated greater resistance to degradation compared to the conventional bulk-fill composite tested. In contrast, the mechanical properties of the conventional composite were more negatively affected by the ultra-fast curing protocol, highlighting the material-dependent response to high-irradiance, short-duration curing.

## Data Availability

Data sets used in the current study are available from the corresponding author upon reasonable request.

## Footnotes

1 R Core Team (2023). R: A language and environment for statistical computing. R Foundation for Statistical Computing, Vienna, Austria. URL https://www.R-project.org/.
