# Correction: Performance of RAFT-based and conventional bulk-fill composites cured with conventional and high irradiance photocuring: a comparative study

**DOI:** 10.1186/s12903-025-07040-9

**Published:** 2025-10-21

**Authors:** Noura Arafa, Dalia I. Sherief, Lamia M. Elmalawany, Mohamed S. Nassif

**Affiliations:** 1 Biomaterials Department, Faculty of Dentistry, Ain-Shams University, African Union Organization Street, Cairo, 11566 Egypt; 2 Badya University, Badya City, Giza, Egypt

**Correction: BMC Oral Health 25, 714 (2025)**

**https://doi.org/10.1186/s12903-025-06046-7**

In this article [1], the author’s name Lamia M. Elmalawany was incorrectly written as Lamia M. Elmalawanya.

Incorrect author name:

Lamia M. Elmalawanya

Correct author name:

Lamia M. Elmalawany
